# A roadmap for understanding sulfadoxine-pyrimethamine in malaria chemoprevention

**DOI:** 10.1017/S0031182025000071

**Published:** 2025-02

**Authors:** Thiery Masserey, Lydia Braunack-Mayer, R Scott Miller, Jörg J Möhrle, Melissa A Penny

**Affiliations:** 1 Swiss Tropical and Public Health Institute, Allschwil, Switzerland; 2 University of Basel, Basel, Switzerland; 3 Gates Medical Research Institute, Cambridge MA, USA; 4 The Kids Research Institute Australia, Nedlands, WA, Australia; 5 Centre for Child Health Research, University of Western Australia, Crawley, WA, Australia

**Keywords:** malaria, mechanisms of action, prevention, resistance, sulfadoxine-pyrimethamine

## Abstract

Sulfadoxine-pyrimethamine (SP) is the standard of care for *Plasmodium falciparum* malaria chemoprevention among pregnant women, infants and children. Developing alternative chemoprevention products and other prevention products, such as vaccines and monoclonal antibodies, requires significant investment. However, knowledge gaps surrounding the activity of SP and resistance put these investments at risk. Therefore, we reviewed SP’s combined antimalarial action, including the individual antiplasmodial components, other antimicrobial effects, impact on malaria immunity development and continued effectiveness in settings with high SP resistance. We created a roadmap of non-clinical and clinical evidence to better understand the effectiveness of SP for chemoprevention and inform the development of new prevention tools.

## Graphical abstract

**Figure:**
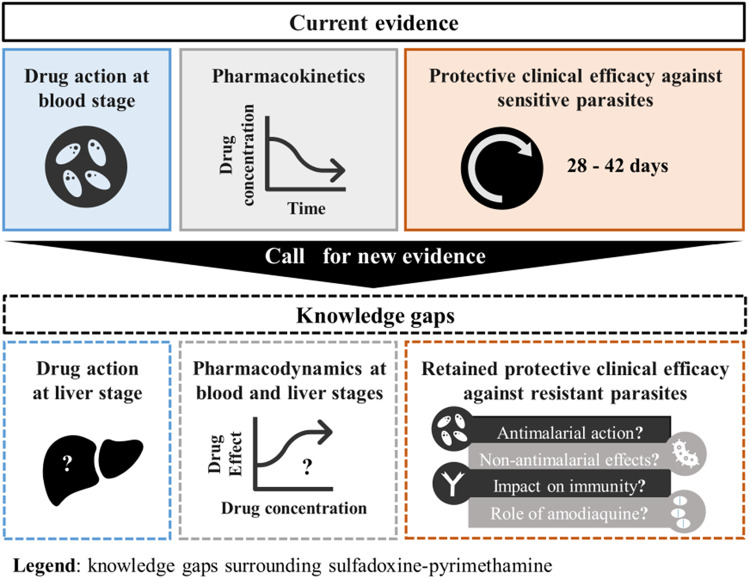

## Introduction

Malaria remains a global health priority. Despite the widespread use of insecticide treated nets, chemoprevention and artemisinin-based combination therapy, the World Health Organization (WHO) estimated 249 million cases and 608 000 malaria-related deaths in 2022 alone (World Health Organization, 2022a). Over 95% of this burden occurs in the African Region, with *Plasmodium falciparum* malaria being the most prevalent and severe.

Sulfadoxine-pyrimethamine (SP) or SP combinations, such as SP-amodiaquine (SP-AQ), are the standard of care for malaria chemoprevention in Africa. SP is active against successive enzymes of the folate synthesis pathway that are essential for the synthesis of parasite DNA and parasite replication in hepatocytes and red blood cells. SP is widely used for malaria chemoprevention due to its low cost (White et al., 2011), safety when given intermittently, and long protection window against malaria (Table 1).

**Table 1.** Summary of protective efficacy or effectiveness of SP for chemoprevention in infants, children, and during pregnancy

Intervention	Type of study	Location of study	Year of collection	Key result	Source
SMC with SP-AQ	Observational study	Burkina Faso, Chad, The Gambia, Guinea, Mali, Niger, and Nigeria	2015–2016	SMC prevented a mean 88·2% (95% CI 78·7–93·4) of clinical cases over 28 days after each cycle of SMC	(ACCESS-SMC Partnership, 2020)
				High prevalence (75% (95% CI 70–79) in 2016) of quadruple mutant	
SMC with SP-AQ	Household-randomized clinical trial	Burkina Faso and Mali	2014–2016	SMC prevents 78·3% (95% CI 76·8–79·6) of clinical cases of malaria in the 28 days after each cycle of SMC	(Cairns et al., 2020)
				High frequency (80% (95% CI 73–79) in 2016) of quadruple mutant	
SMC with SP-AQ	Non-randomized controlled trial	Uganda	2021	SMC prevented 92% (95% CI 90·0–94·0) of clinical cases among children during the 5-month study period. The prevalence of molecular markers was not reported, but researchers assumed a high prevalence of quintuple mutants in the region	(Nuwa et al., 2023)
SMC with SP-AQ	Mathematical modelling study	Archetypal modelled setting with seasonal malaria transmission	No data collected	Effectiveness of SMC with SP-AQ will decrease with the spread of the quintuple mutant in West Africa, but considerable effectiveness will remain	(Masserey et al., 2024)
PMC* with SP	Cluster-randomized, placebo controlled clinical trial	Ghana	2000–2004	Post-PMC, SP provides 42 days of protection against clinical malaria in Ghana	(Cairns et al., 2008)
				Prevalence of molecular markers not reported but assumed to be low in the region based on other studies (e.g. see the study below, which reported prevalence in Ghana)	
PMC* with SP	Mathematical modelling study	Data sourced from 7 randomized placebo controlled trials in Gabon, Ghana, Mozambique and Tanzania	1999–2008	Duration of protection provided by SP post-PMC against clinical malaria decreased in settings with higher degrees of resistance	(Griffin et al., 2010)
				Length of protection was equal to 42 days in Ghana (quintuple mutant absent from the population) and 21 days in Tanzania (frequency of quintuple mutant of 89–2%)	
IPTp with SP	Prospective, single-arm clinical trial	Burkina Faso, Kenya, Malawi, Mali, Uganda, Zambia	2009–2013	Median time before a pregnant women received ITPp and developed a patent blood stage infection was reduced in settings with higher degrees of resistance	(Desai et al., 2016)
				Length of protection post-IPTp was 42 days in areas with low (<1%)frequency of quintuple mutant and 21 days in areas with high frequency (>95%) of quintuple mutant	
IPTp with SP	Review	Multiple study sites across Africa	1993–2020	The protective effectiveness of SP against malaria infection decreases with higher degrees of resistance	(Gutman et al., 2022)
				In areas of high resistance (sextuple mutant prevalence >5%), SP did not seem to confer protection against malaria infection	
				SP continued to reduce the risk of maternal anaemia (relative risk reduction of 8·2%) and improve children’s birthweight (relative risk reduction of 16%) in the highest SP resistance areas (sextuple mutant prevalence >5%).	
SMC with SP-AQ, PMC* with SP, and IPTp with SP	Review	Multiple study sites across Africa and Asia	No data collected	For SMC, evidence for reduced SP-AQ effectiveness with increasing degrees of resistance to SP is limited, due to the paucity of data	(Plowe, 2022)
				For PMC and IPTp, there is some evidence that supports a finding of reduced SP effectiveness against morbidity with increasing degrees of resistance to SP	

* Previously referred to as intermittent preventive treatment in infants (IPTi). AQ: amodiaquine; IPTp: intermittent preventive treatment in pregnancy; PMC: perennial malaria chemoprevention; SMC: seasonal malaria chemoprevention; SP: sulfadoxine-pyrimethamine.

There are several high risk populations targeted for malaria chemoprevention strategies that are used for their cost-effectiveness and public health impact (White et al., 2011). In intermittent preventive treatment in pregnancy (IPTp), SP is given to pregnant women at scheduled intervals from their second trimester, regardless of whether they are infected with *P. falciparum* (World Health Organization, 2023). This approach reduces malaria incidence in pregnant women and their infants, and improves birth outcomes by reducing the risk of low birth weight and foetal anaemia (Gutman et al., 2022). IPTp is not recommended in the first trimester due to safety concerns, although evidence from observational cohort studies suggests that concerns may be unfounded (Phillips-Howard et al., 1998; Mosha et al., 2014).

In perennial malaria chemoprevention (PMC), SP is given to infants from 3 months of age in areas with year-round transmission, at intervals aligned with routine healthcare visits (World Health Organization, 2023). While uptake of this intervention has been limited to date, it is effective in reducing the incidence of clinical malaria, severe malaria, and anaemia in its target population (Plowe, 2022). More recently, in 2023, the WHO recommended that PMC be given to children beyond 12 months of age and highlighted the need to evaluate effectiveness beyond 24 months (World Health Organization, 2023).

SP is also used for seasonal malaria chemoprevention (SMC) in combination with a 3-day course of AQ to protect children in regions with moderate-high malaria transmission. This intervention delivers SP-AQ to children at monthly cycles across the malaria season, protecting them against clinical disease and hospitalization during this high-risk period (World Health Organization, 2023). SMC has been adopted by 17 countries in sub-Saharan Africa and is administered to almost 49 million children per cycle (World Health Organization, 2022a). SMC mainly targets children between 3-months and 5-years old, but some countries have extended this to children under 10. SP-AQ is also used in some countries for chemoprevention in school-aged children between 5 and 15 years old. However, it is still uncertain whether children older than 10 should be targeted by a chemoprevention program that uses SP-AQ, due to the risk of first trimester pregnancy among female recipients and the lack of safety data (White et al., 2011).

Additionally, recent WHO recommendations support new populations targeted for chemoprevention, such as in post-discharge malaria chemoprevention for 4–6 months to allow a child to fully recover from a severe anaemia episode (World Health Organization, 2023).

SP was originally approved in 1981 as a single dose antimalarial treatment in regions with chloroquine-resistant *P. falciparum* (Centers for Disease Control and Prevention, 1982). The use of SP for treatment has been discontinued in many countries due to the presence of drug-resistant parasites, which reduced its treatment efficacy (World Health Organization, 2020). *P. falciparum* parasites with multiple mutations in the *dihydropteroate synthase (dhps)* and *dihydrofolate reductase (dhfr)* genes have reduced sensitivity to sulfadoxine and pyrimethamine, respectively (Cowman et al., 1988; Peterson et al., 1988; Zolg et al., 1989; Brooks et al., 1994; Wang et al., 1997). The prevalence of these mutations varies greatly across Africa (Okell et al., 2017; ACCESS-SMC Partnership, 2020) and the relationship between combinations of mutations and treatment failure has been previously reported by multiple studies. In West Africa, a quadruple mutant parasite (with *dhfr*-N51I, *dhfr*-C59R, *dhfr*-S108N, and *dhps-*A437G mutations) partially resistant to SP (treatment failure: 1·3%–41·1% (Kublin et al., 2002; Staedke et al., 2004; Desai et al., 2016)) is highly prevalent (more than 70%) (ACCESS-SMC Partnership, 2020). A quintuple mutant with an additional mutation *dhps-*K540E (treatment failure: 10%–75% (Kublin et al., 2002; Staedke et al., 2004; Desai et al., 2016)) is also emerging in this region (frequency below 5%) (ACCESS-SMC Partnership, 2020; Mahamar et al., 2022), and is already highly prevalent in East Africa (frequency above 50%) (Okell et al., 2017). Moreover, in East Africa, a sextuple mutant is emerging (Gutman et al., 2015; Bwire et al., 2020), which carries an additional mutation *dhps-*A581G with very high-grade resistance (82·2% treatment failure (Gesase et al., 2009)). In addition, parasites with a low degree of resistance to AQ (with mutations *pfmdr1-*86Y, *pfmdr1-*184Y, *pfmdr1-*1246Y, and *pfcrt*-76T) (Picot et al., 2009; Venkatesan et al., 2014; Arya et al., 2021) are present in multiple regions across Africa overlapping with regions of SP-resistance (Ehrlich et al., 2021), potentially further challenging SMC efforts.

Massive investments are being made to develop new tools in response to gaps in the existing malaria prevention toolkit, as well as to concerns that further acquisition of resistance to SP may erode the protective effectiveness of SP and SP-AQ. Novel treatment and preventive tools include new oral drug combinations, long-acting injectables (Burrows et al., 2017), monoclonal antibodies (Aleshnick et al., 2022) and CSP-based malaria vaccines. Some of these tools – in the case of RTS,S/AS01 and R21 vaccines – are being trialled in combination with SMC (Datoo et al., 2021; Cairns et al., 2022, 2024). However, recent studies suggest that, despite a high degree of antiplasmodial resistance, chemoprevention programs using SP or SP-AQ are still effective in improving clinical incomes; the duration of protection conferred by SP against clinical malaria decreases with increasing degrees of resistance to SP, but some general health benefits seem to be retained even against the sextuple mutant (Table 1). This may be explained by the fact that the use of SP for treatment depends solely on the ability of SP to cure a high-density blood stage infection. In contrast, the use of SP for chemoprevention depends on the ability of SP to prevent health burdens.

In the haste to find alternatives to SP and SP-AQ, insufficient time and resources may have been invested into fully understanding the way SP works to prevent health burdens. Here, we review literature and clinical trial data to identify the full spectrum of activity for SP and SP-AQ. We report substantial knowledge gaps regarding the liver and blood stage activity of SP, the impact of SP on malaria immunity acquisition, and the role of AQ to the protective effectiveness of SMC. We also discuss the role of the antimicrobial and anti-inflammatory activity of SP, referring to the drug combination’s ability to kill or inhibit the growth of bacteria and reduce inflammation.

As a result of these gaps, it is not fully known how SP and SP-AQ contributes to the observed clinical benefits of malaria chemoprevention in the face of resistance. As discussed in this paper, these knowledge gaps prevent an accurate and fair comparison between SP or SP-AQ and alternative chemoprevention tools, which ultimately prevents informed decisions to prioritize investment and anticipate when the deployment of SP or SP-AQ should be stopped. We have developed a roadmap for understanding the contribution of SP to malaria chemoprevention. We call on funders, drug developers, researchers, regulatory agencies and policymakers to generate new and essential evidence for this old drug combination, which is a crucial step in successfully guiding the development of new malaria preventive tools.

## The preventive activity of SP

*P. falciparum* has a complex life cycle and tools that target this parasite within the human host can be divided into two categories. Anti-infective tools target sporozoites delivered by the mosquitoes or parasites infecting the liver. Blood-stage tools target the parasites once they emerge from the liver stage into the bloodstream and infect red blood cells. Here we first review the antiplasmodial activity of SP in these two categories. Then, we discuss how AQ contributes to the antiplasmodial effects of SP. We then discuss the antimicrobial and other activities of both SP and AQ. Finally, we review what is known about the impact of SP and SP-AQ on the development of blood-stage immunity.

### Anti-infective activity of SP

The anti-infective activity of SP is limited to the liver stage of *P. falciparum* infection. However, little is known about the liver stage activity of SP. An *in vitro* study has demonstrated that pyrimethamine can kill rodent malaria parasites (*P. yoelii*) infecting human hepatocyte cells (HepG2 cells) (Delves et al., 2012). Friesen and others have shown that mutations conferring resistance to the blood stage action of pyrimethamine also reduce the liver stage activity of pyrimethamine against *P. berghei* in mouse models (Friesen et al., 2011). No published clinical trial has reported the action of pyrimethamine on the liver stage of *P. falciparum* in humans, estimated the duration of this effect, or explored how liver stage activity is affected by *dhfr* gene mutations. In contrast to pyrimethamine, sulfadoxine did not impact rodent malaria parasites (*P. yoelii*) infecting HepG2 cells *in vitro* (Delves et al., 2012), and thus may not affect the liver stage of *P. falciparum* in human. However, it is not known whether sulfadoxine can enhance the action of pyrimethamine on liver stage parasites.

### Blood stage activity of SP

Many studies have identified pharmacokinetic (PK) properties of SP in infants (Salman et al., 2011; de Kock et al., 2018), children (Bell et al., 2011; Tekete et al., 2011; de Kock et al., 2018) and in pregnant women (Green et al., 2007; Karunajeewa et al., 2009; Nyunt et al., 2010; de Kock et al., 2017). Physiologically-based pharmacokinetics models, which consider more detailed physiological information than PK models (such as organ characteristics), are available but have not yet been applied to support PK analyses in vulnerable populations, such as pregnant women (Abla et al., 2023).

Several studies have explored the clinical impact of SP on the blood stage of drug-sensitive parasites and identified combinations of *dhfr* and *dhps* gene mutations that cause treatment failure when SP is used as a treatment (and not as a preventive tool) (Cowman et al., 1988; Brooks et al., 1994; Kublin et al., 2002; Staedke et al., 2004; Gesase et al., 2009; Desai et al., 2016). Older studies have also identified the antiplasmodial clinical efficacy of sulfadoxine and pyrimethamine as treatment in monotherapy and in combination (Hererro, 1966; Laing, 1966; Lucas et al., 1969; Snyder et al., 2007). Researchers have also conducted controlled human malaria infections to estimate the parasite reduction ratio and parasite clearance half-life of drug-sensitive parasites following treatment with SP (Marquart et al., 2015).

Sulfadoxine and pyrimethamine are known to have a synergistic effect on the blood stage of the parasite (Hererro, 1966) when used together. *In vitro* studies have reported that this synergistic effect is retained against pyrimethamine-resistant parasites (Brockelman and Tan-Ariya, 1982; Eastham and Rieckmann, 1983; Chulay et al., 1984). Sulfadoxine has also been shown to enhance the activity of pyrimethamine against the quintuple mutant (Bwijo et al., 2003). Another study has also shown that, for parasites with the mutation combinations *dhfr*-N51I/S108N/164L and *dhps-*A437G/A581G or *dhfr*-N51I/S108N/164L and *dhps-* A437G/K540E/A581G, the effect of both drugs was additive instead of synergistic (Bacon et al., 2009).

However, *in vitro* data reporting synergistic effects against the quadruple, quintuple and sextuple mutants are limited. Thus, it is challenging to build a comprehensive pharmacodynamic (PD) model that could predict the duration of the protection conferred by SP post-treatment against each genotype. To the best of our knowledge, only Htay and colleagues have developed a PD model that considers the SP’s synergistic effect on drug-sensitive parasites (Htay et al., 2020). This model is based on the work of Gatton and colleagues (Gatton et al., 2004), which estimated the probability of parasite survival of drug-sensitive parasites at different concentrations of SP based on *in vitro* data. Gatton and colleagues (Gatton et al., 2004) also estimated the probability of survival for different combinations of mutations but had to perform some extrapolation due to the limited availability of data. Thus, additional data are needed to build a comprehensive PD model against each resistant genotype.

### Contribution of amodiaquine (AQ) in SMC with SP-AQ

AQ is a 4-aminoquinoline active against *P. falciparum* blood stage infections, historically used as an alternative to chloroquine (Olliaro et al., 1996; White, 1996), and currently used in combination with artesunate for the treatment of uncomplicated malaria (World Health Organization, 2023). SP is combined with AQ for SMC to ensure that infections are cleared rapidly when SMC is deployed. AQ also provides a duration of protection against infection that varies from 10·2 to 18·7 days, depending on the presence of parasites with a low degree of resistance to AQ (Bretscher et al., 2020).

Recent studies that have implemented SMC with SP-AQ in East Africa, where the quintuple mutant has a high prevalence (above 60% frequency) and parasites are sensitive to AQ (Molina-de la Fuente et al., 2023; Baker et al., 2024), have reported that SMC remains highly effective (Nuwa et al., 2023). However, it is not known whether the effectiveness of SMC is mainly driven by the prophylactic action of AQ or the remaining effect of SP on the quintuple mutant (see Table 1). Consequently, it is not known whether AQ would maintain the effectiveness of SMC in regions with sextuple mutants. It is also unknown how low adherence to the 3-day AQ regimen and resistance to AQ could influence the effectiveness of SMC.

### Antimicrobial and other activities of SP and AQ

The continued benefits of SP in ITPp despite resistance may come from the antimicrobial action of sulfadoxine. A recent review highlighted that, in areas with a high degree of resistance (defined as a prevalence of the sextuple mutant above 5%), the ability of IPTp with SP to prevent or clear *P. falciparum* infection was greatly diminished (Gutman et al., 2022). Nevertheless, ITPp continued to reduce the risk of maternal anaemia in pregnancy and to improve the birthweight of children (Gutman et al., 2022). Recent clinical studies have reinforced that SP continues to reduce the frequency of adverse pregnancy outcomes in areas with a high degree of resistance to SP (8%–40% frequency of sextuple mutant) (Madanitsa et al., 2023). Studies have also indicated that the benefit provided by SP on birthweight is mediated by the ability of the drug combination to promote maternal weight gain during the 2nd and 3rd trimesters (Waltmann et al., 2022). This may be due to the impact of antimicrobial activity of sulfadoxine on the maternal gut microbiome (Waltmann et al., 2022). Or, it could be from the ability of sulfadoxine to reduce the risk of bacterial infections, such as *Gardnerella vaginalis, Staphylococcus aureus, Streptococcus pneumoniae* (Capan et al., 2010). In addition, one study reported that IPTp with SP reduced the impact of sexually transmitted infections such as *Neisseria gonorrhoeae* and *Chlamydia trachomatis* on adverse birth outcomes (Chico et al., 2017). Sulfadoxine may also improve infant birth weight by modifying the relationship between inflammation and adverse outcomes (Cheng et al., 2024), thus allowing better placental vascular development (Unger et al., 2019).

These theories are, however, complicated by the results of recent findings that assess the efficacy of alternative drug combinations for IPTp. One study found that the combination of azithromycin and chloroquine, an antibiotic and antimalarial, was not superior to SP against pregnancy outcomes in a multi-centre study in areas with SP resistance (Kimani et al., 2016). A more recent study found that dihydroartemisinin-piperaquine (an antimalarial) with and without azithromycin was not better than SP in reducing adverse outcomes during pregnancy despite a better antiplasmodial effect (Madanitsa et al., 2023). These studies suggest that SP has benefits beyond its antiplasmodial and antimicrobial properties.

While these studies focus on IPTp, one can hypothesize that the additional antimicrobial effects of SP may also play a role in SMC and PMC. Some malarial fevers may arise only due to co-infections of *P. falciparum* with other pathogens and would not occur without co-infections. If the other antimicrobial effects of SP reduce co-infections with other pathogens during SMC and PMC, SP could decrease the likelihood that a malaria infection leads to a malaria fever.

Recent attention has also been drawn to novel activities for AQ. For example, AQ is active against autoimmune diseases, cancers, neurodegenerative diseases (Kim et al., 2017) and chronic inflammatory diseases (Oh et al., 2016). Little is known about whether these other benefits contribute to the clinical effectiveness of SMC with SP-AQ.

### Impact of malaria interventions on immunity acquisition

Individuals repeatedly exposed to the parasite gradually acquire partial immunity that can prevent the symptoms of malaria. Immunity can be developed against parasites at the different stages of its cycle within the host (e.g. sporozoites, asexual blood stages, gametocytes). Immunity developed at the blood stage has a key role in reducing the parasite density and severity of the symptoms (Mandala et al., 2021).

All interventions that prevent blood stage *P. falciparum* infection (such as a pre-erythrocytic vaccine) may change the natural course of the acquisition of blood stage immunity to *P. falciparum* (Cairns et al., 2015). However, if the protective effect of SP is mainly driven by an imperfect liver-stage activity, some parasites may complete the liver stage and be released into the bloodstream. Nevertheless, the resulting blood-stage infection may start at a lower density, which may allow more time for a boost to blood-stage immunity that could contribute to controlling infection and reducing symptoms. Similarly, if the protective effect of SP is mainly driven by an imperfect blood-stage activity that slows parasite growth, a similar delay may allow more time for the development of immunity. Such blood-stage immunity could also further prevent clinical cases during following infections even once SP no longer protects against infection (World Health Organization, 2022b).

However, studies examining the impact of SP or SP-AQ on immunity acquisition have reached conflicting conclusions. For example, some studies have reported that children receiving SP-AQ through SMC develop lower concentrations of antibodies against blood stage malaria (Ndiaye et al., 2015; Mahamar et al., 2017), but more recent evidence reports an opposite trend (Mahaman Moustapha et al., 2021). As there are as of yet no validated biomarkers for blood stage immunity, this represents a challenge for understanding the impact of SMC on the development of blood stage immunity.

## Knowledge gaps and their implications for developmental and regulatory approval of new prevention tools

With SP or SP-AQ established as cornerstones of malaria prevention, the lack of knowledge regarding the full spectrum of activity of SP and SP-AQ has become critical. The review highlighted multiple knowledge gaps, of which five key gaps are listed in Box 1.

Box 1. Knowledge gaps regarding the activity of SP and SP-AQ

**Knowledge gaps**

1. There are limited PD data and models available to simulate the synergic antiplasmodial blood stage action of SP on resistant *P. falciparum* parasites such as the quadruple, quintuple and sextuple mutant.
2. There is no clear understanding of SP’s action against the liver stage of *P. falciparum*, particularly for parasites with mutations in *dhfr and dhps* genes.
3. Little is known about the impact of SP and SP-AQ on the acquisition of blood stage immunity to malaria and the extent to which this impact affects chemoprevention effectiveness.
4. The extent to which the effectiveness of SP is potentially driven by its other antimicrobial activities is not fully understood, including its:

- Impact on the host microbiome.
- Antimicrobial activity.
- Impact on systemic inflammation.
- Indirect effects on malarial outcomes due to reductions in comorbidities, particularly in promoting maternal weight gain through IPTp and in clearing co-infections in infants and children.

1. The extent to which AQ contributes to the benefits provided by SMC in settings with high SP resistance is not fully understood.

Table 2 outlines these knowledge gaps together with a list of the pre-clinical and clinical evidence required to better understand the effects of SP and SP-AQ.

These knowledge gaps in the activities of SP and SP-AQ (antiplasmodial, other antimicrobial activity and impact on malaria immunity development) will continue to hamper progress in malaria prevention. First, this lack of understanding prevents us from comprehensively comparing SP or SP-AQ to new chemoprevention candidates at different stages of development. As discussed, there is limited *in vitro* and *in vivo* PD data for SP at the liver and blood stages. This limits the ability to build a PD model, which is needed to enable assay translation and benchmarking to the standard of care for new drug candidates (Hughes et al., 2021).

Second, developers of new prevention tools, including long-acting injectables, monoclonal antibodies and vaccines, will need to run clinical trials that compare the effectiveness of their tools to SP or SP-AQ. It is thus essential to better understand the activity of SP and SP-AQ, to make a fair comparison between the standard of care and a new prevention tool. Appropriate clinical endpoints that accurately assess the ability of tools to prevent both malaria-specific outcomes and general health outcomes must be understood and agreed on. A study that only measures endpoints related to malaria health outcomes would miss the impact of SP on the general health benefits potentially provided by SP (such as through antimicrobial activity or other indirect benefits).

Third, the lack of clarity around the antiplasmodial liver stage action and other antimicrobial effects of SP limit the ability to accurately parameterize the effect of SP or SP-AQ in mathematical models. Mathematical models, which can link the characteristics of a particular intervention, population or setting with the likely public health outcome, have been used throughout the malaria product development lifecycle. Modelling based on imperfect assumptions of the activity of SP (including immunity development and antimicrobial activity) may result in unfair comparisons to preventive interventions with respect to both their expected public health impact and cost-effectiveness.

Finally, uncertainty around the actions of SP and SP-AQ limits the ability to make informed product prioritization and investment decisions, since we do not know yet which properties preventive tools need to have to perform as well as SP in terms of clinical outcomes. For example, without more knowledge of the liver-stage or other antimicrobial activities of SP, it is not known whether these mechanisms are important; it is not known if they should be looked for at the initial development stages of new chemoprevention tools and captured in cost-effectiveness studies. If the antimicrobial effects are important, should SP be dosed in combination with *Plasmodium*-targeted prevention, such as with vaccines or monoclonal antibodies? These knowledge gaps may lead to the development of inappropriate drugs, missed opportunities and a waste of resources, as we may realize that the new product is missing an essential property at a late stage of development.

## Roadmap to understanding SP

As investment in prevention tools increases in response to the threat of SP-resistance, the need for additional evidence regarding the action of SP has become urgent. Thus, Table 2 describes the pre-clinical and clinical evidence required to better understand the full effects of SP and SP-AQ and fill the knowledge gaps described in Box 1. The WHO recently highlighted the need for additional studies to explore the effect of SP on pregnancy outcomes for IPTp (Gutman et al., 2022). Generating this evidence will require a commitment of funding, resources, and coordination.

**Table 2.** Pre-clinical and clinical evidence needed to better understand the effects of SP and SP-AQ

Knowledge gap	Intervention(s)	Evidence required to address knowledge gap
Synergistic antiplasmodial blood stage activity of SP	IPTp, PMC, SMC	*In vitro* study reporting the synergetic effect of SP against quadruple, quintuple, and sextuple mutants.
		Blood stage challenge study assessing the prophylactic period conferred by SP against each mutant to elucidate how strongly the blood activity of SP contributes to the protective effectiveness of SP.
Liver stage activity of SP	IPTp, PMC, SMC	Sporozoite challenge against sensitive and resistant parasites with an ultrasensitive qRT-PCR endpoint
Impact of SP and SP-AQ on acquisition of blood stage immunity	PMC, SMC	Observational study of natural exposure to malaria: an assessment of entomological, parasitological, and clinical data in children of different age groups in different transmission intensities, including serological endpoints (such as concentration of antibodies against blood stage antigens) to assess the exposure to malaria.
		Challenge human malaria infection or clinical efficacy trial that assesses the ability of SP/SP-AQ to prevent blood stage infection using ultrasensitive RT-PCR to monitor breakthrough infections
		Systematic review or clinical study to evaluate SP/SP-AQ consequences on age patterns of disease and post-intervention effects such as risk of rebound
		Modelling studies that monitor SP/SP-AQ’s impact on immunity development, chemoprevention outcomes under various drug mechanisms of action
Other antimicrobial effects of SP	IPTp	*In vitro* and *in vivo* studies of SP’s impact on gestational weight gain and on maternal gut microbiome
	IPTp, PMC, SMC	Systematic reviews on the impact of SP’s other antimicrobial activities on outcomes in IPTp, PMC, and SMC
		Clinical trial or observational study including non-parasitological and non-clinical malaria endpoints to capture indirect effects on both general and malaria health outcomes and screening for common bacterial pathogens
AQ’s contribution to SP-AQ’s effectiveness	SMC	Clinical trial with comparator arms of SP-AQ, SP monotherapy, and AQ monotherapy in areas with a high degree of resistance to SP. Such a study should be run in a region that does not currently implement SMC to be ethical.
SP’s safety in pregnancy	IPTp	Systematic review of SP’s safety in the first trimester of pregnancy
	SMC	Define the maximum age group that can be targeted by SMC with SP-AQ based on updated safety data

AQ: amodiaquine; IPTp: intermittent preventive treatment in pregnancy; PMC: perennial malaria chemoprevention; SMC: seasonal malaria chemoprevention; SP: sulfadoxine-pyrimethamine.

In order to produce the evidence required to fill the identified knowledge gaps (Table 2), a transparent and efficient pathway for the regulatory approval of new malaria prevention products should be defined now. This will require that normative agencies, regulatory agencies and developers define the essential pre-clinical and clinical evidence required for new preventative tools where SP or SP-AQ is the standard of care. This evidence must consider both the antiplasmodial and other antimicrobial effects of SP and requires that appropriate clinical endpoints are defined to assess these effects. For policy recommendations for new preventive tools, consensus is also needed on when and how to evaluate the relative cost-effectiveness of a new intervention compared with SP or SP-AQ.

## Conclusion

SP and SP-AQ remain the most cost-effective tools for malaria prevention among children and pregnant women. The available evidence for the full spectrum of activity of SP and SP-AQ has been reviewed, highlighting knowledge gaps regarding the liver and blood stage antiplasmodial activity of SP, its other antimicrobial effects, its impact on malaria immunity acquisition, and the contribution of AQ to the protective effectiveness of SMC (as summarized in Box 1). With substantial resources being invested in developing new prevention tools, the need to generate evidence to address the knowledge gaps (as described in Table 2) is urgent. Therefore, policy decision-makers must articulate the minimum requirements needed for novel interventions to be recommended as a replacement or addition to SP or SP-AQ. Should these knowledge gaps remain, precious resources may be wasted in malaria prevention simply because the standard of care is not adequately understood.
